# Effect of Environmental Variation on Estimating the Bacterial Species Richness

**DOI:** 10.3389/fmicb.2017.00690

**Published:** 2017-04-19

**Authors:** Yongjian Chen, Jialiang Kuang, Pu Jia, Marc W. Cadotte, Linan Huang, Jintian Li, Bin Liao, Pandeng Wang, Wensheng Shu

**Affiliations:** ^1^State Key Laboratory of Biocontrol, Guangdong Key Laboratory of Plant Resources and Conservation of Guangdong Higher Education Institutes, College of Ecology and Evolution, Sun Yat-sen UniversityGuangzhou, China; ^2^Department of Biological Sciences, University of Toronto Scarborough, TorontoON, Canada; ^3^Department of Ecology and Evolutionary Biology, University of Toronto, TorontoON, Canada

**Keywords:** richness estimation, non-parametric estimators, environmental variation, environmental heterogeneity equation, forest dynamic plot

## Abstract

Estimating the species richness of microorganisms is of great importance in predicting, maintaining and managing microbial communities. Although the roles of environmental heterogeneity and geographical distance in structuring soil microbial communities have been studied intensively, the effects of environmental and spatial variation on the species richness estimation have not been examined. To this end, we have explored their effects on estimating the belowground soil bacterial species richness within a 50 ha forest dynamic plot (FDP) using a published massive sequencing dataset with intensive sampling scheme. Our resampling analyses showed that, for a given sequencing depth, increasing the sample size could significantly enhance the detection of rare species by capturing more of the environmental and spatial variation, thus obtaining higher observed and estimated species richness. Additionally, the estimates of bacterial species richness were significantly and positively correlated with environmental variation among samples, indicating that environmental filtering was the main mechanism driving the processes of community assembly for belowground soil bacterial communities in the plot. Moreover, this effect of environmental variation could be markedly alleviated when the sample size was higher than 450, and thus we predicted that there were at least 42,866 soil bacterial species based on 8,296,878 sequences from 550 samples in the whole 50 ha FDP. Furthermore, we built a power law environmental heterogeneity equation (EHE) as a decision-tool to determine an approximate sample size for comprehensively capturing the environmental gradient within a given habitat. Collectively, this work further links the inherent environmental and spatial variation to the estimation of soil bacterial species richness within a given region, and provides a useful tool of sampling design for a better understanding of microbial biogeographic patterns and estimation of microbial biodiversity.

## Introduction

Species richness is a fundamental property of an ecological community, and further is essential for pattern prediction, ecological modeling and theory test in community ecology (MacArthur and Wilson, 1967; Connell, 1978; Stevens, 1989; Hubbell, 2001). Given the profound importance of species richness in sustaining ecosystem function (Maestre et al., 2012) and addressing urgent issues in conservation biology (Groom et al., 2006), how many species coexist within a given locality or, indeed, on Earth has remained a central theme in ecology (May, 1988). However, to answer this question in an extraordinary diverse natural community, exhaustedly counting every single species is seemingly an endless work. Therefore, model-based estimation of species richness according to the observed abundance and/or incidence data derived from field surveyed samples is necessary. Under this framework, by using the worldwide biological inventories, the number of tree species in tropical region has recently been estimated to fall between 40,517 and 53,345 (Slik et al., 2015), and the mean global estimates of beetles, insects and terrestrial arthropods are 1.5, 5.5, and 6.8 million, respectively (Stork et al., 2015). Nevertheless, little is known about the species richness of microorganisms, which represent a large proportion of Earth’s biodiversity (Whitman et al., 1998) and are the key contributors to ecosystem function (Bell et al., 2005).

Soil is the most important terrestrial habitat on Earth, and soil bacteria play critical roles in driving various ecological processes and biogeochemical cycles (Hogberg et al., 2001; Kowalchuk and Stephen, 2001; Rillig and Mummey, 2006). However, estimating the bacterial species richness is particularly challenging because of their enormous diversity and number of individuals [e.g., soil on Earth contains an estimated 2.6 × 10^29^ prokaryotic cells (Whitman et al., 1998)]. By using culture-independent approaches (e.g., clone library inventory and high-throughput sequencing of 16S rRNA gene), estimating the number of operational taxonomic unit (OTU) at 97% sequence similarity is the most typically and widely used strategy to proximately measure the bacterial species richness (Rosselló-Mora and Amann, 2001; Gevers et al., 2005; but see other similarity thresholds in Yarza et al., 2014), and recent studies have attempted to estimate the number of bacterial OTUs from different soil habitats such as grassland, agriculture land and sediment (Hughes et al., 2001; Curtis et al., 2002; Bohannan and Hughes, 2003; Kemp and Aller, 2004; Hong et al., 2006; Schloss and Handelsman, 2006; Roesch et al., 2007; Youssef et al., 2009). The estimates of bacterial OTU richness across these soil samples range from a few dozens and hundreds (Hughes et al., 2001; Bohannan and Hughes, 2003; Kemp and Aller, 2004) to thousands and tens of thousands (Curtis et al., 2002; Hong et al., 2006; Schloss and Handelsman, 2006; Roesch et al., 2007; Youssef et al., 2009). However, little research has explored the underlying mechanism accounting for this uncertainty on estimating the bacterial OTU richness.

Increasingly, microbial biogeographic studies have documented that both niche-based environmental processes and dispersal-based spatial processes structure soil bacterial assemblages (Horner-Devine et al., 2004; Ramette and Tiedje, 2007; Ranjard et al., 2013), indicating a heterogeneous distribution of bacterial OTUs within a given region. This non-random distribution of bacterial OTUs is largely due to their fitness differences that influenced by the environmental adaptation of soil physicochemical properties such as pH (Fierer and Jackson, 2006) and C/N ratio (Bates et al., 2011), and/or their dispersal limitation that caused by the limited dispersal ability and geographic barriers (Martiny et al., 2011). Although the importance of these two ecological processes in structuring the bacterial distribution patterns have been well studied, their effects on the estimates of bacterial OTU richness are largely unknown, implying that adequately capturing these inherent environmental and spatial variation is helpful to fill this knowledge gap and may obtain a more reliable estimate of total bacterial species richness within a given region (i.e., gamma diversity). However, previous estimates are mainly the quantification of bacterial alpha diversity with only a single (Schloss and Handelsman, 2006; Roesch et al., 2007) to dozens of samples (Kemp and Aller, 2004), and this insufficient sampling makes the reliable estimation of gamma diversity for a given area an impossible task. Additionally, the species abundance distribution curves of bacterial communities usually show a pattern that a steep slope upward to the left (Hong et al., 2006; Bunge et al., 2014), indicating that a large fraction of OTU richness is contributed by rare OTUs with low abundance. However, most of these rare OTUs can only be detected occasionally in a few samples even if high-throughput sequencing strategies are applied in diversity surveys of microbial communities (Nemergut et al., 2011). This implies much stronger effects of environmental and spatial variation on the distribution of these rare species, emphasizing the necessity of intensive sampling scheme for the accurate estimation of bacterial gamma diversity.

Here, to explore the effects of environmental and spatial variation on estimating soil bacterial OTU richness, we reanalyzed the published massive 16S rRNA gene (V4 hypervariable region) sequencing dataset of belowground soil bacterial communities in a 50 ha FDP located in Barro Colorado Island (BCI), Panama (Barberán et al., 2015), and performed three resampling analyses to show their effects by statistically controlling the sequencing depth and/or sample size (see detailed methods below). Specifically, 550 soil samples that uniformly distributed throughout the plot were previously collected with detailed records of the soil environmental properties and the spatial coordinates. This intensive sampling design comprehensively captures the environmental and spatial variation in this plot, making it an ideal system for addressing our scientific questions. Our results indicated that the observed and estimated soil bacterial gamma diversity were significantly affected by environmental variation among samples. While the intensive sampling scheme could markedly alleviate this effect, providing a more reliable estimate of soil bacterial gamma diversity in the 50 ha plot. Furthermore, we built an environmental heterogeneity equation (EHE) according to the power law function, *E* = *cS^z^*, to describe the relationship between the sample size (*S*) and the captured environmental gradient (*E*), and demonstrated that this equation is a useful decision-tool for sampling design.

## Materials and Methods

### Data Collection

Among the 63 FDPs within a global monitoring network called the Center for Tropical Forest Science-Forest Global Earth Observatory (CTFS—ForestGEO)^1^, the BCI plot is the first established FDP and the aboveground plant species richness, spatial distribution of stems and soil nutrients in this plot have been studied intensively (Condit et al., 2000; John et al., 2007). It has been reported that there are approximately 258 plant species for all trees ≥ 1 cm diameter at breast height (DBH) in the 50 ha BCI plot (John et al., 2007). A recent study has further found that the taxonomic and phylogenetic beta diversities of belowground soil microbial communities were strongly correlated with those of aboveground plant communities in this plot (Barberán et al., 2015). The massive sequencing dataset in this study has provided a comprehensive profile of belowground soil microbial communities and it is publicly available in FigShare^2^.

Detailed information about sampling scheme, sequence processing and methods of measuring soil environmental variables were described previously (Barberán et al., 2015). The pH value of the soil samples vary from 4.6 to 7.6, and a wide range of gradient was also found for other environmental variables in this plot (Supplementary Table S1). A total of 573 samples were sequenced to assess the belowground soil bacterial communities, the number of bacterial sequences per sample varies several orders of magnitude, from 3 to 29,953. Therefore, to reduce the potential sequencing biases among samples, samples with extremely low or high number of sequences were discarded and then leaving a total of 550 samples for the subsequent analyses. As a consequences, 8,296,878 bacterial quality sequences were generated by the Illumina Miseq platform, with a range of 8,434 to 21,531 sequences per sample.

### Selection of Best Estimators of Soil Bacterial OTU Richness

A series of non-parametric and parametric estimators were used to estimate bacterial OTU richness. In our analyses, abundance-based non-parametric estimators included Chao1 (Chao, 1984) and abundance-based coverage estimator (ACE; Chao and Lee, 1992), while incidence-based non-parametric estimators included Chao2 (Chao, 1987) and incidence-based coverage estimator (ICE; Lee and Chao, 1994). Jackknife1 (Burnham and Overton, 1979) and Jackknife2 (Burnham and Overton, 1979) non-parametric estimators could be calculated by using both abundance-based (Jackknife1_ab_ and Jackknife 2_ab_) and incidence-based (Jackknife1_in_ and Jackknife2_in_) data. All these non-parametric estimators and their corresponding 95% confidence intervals (CI) were calculated using the Species Prediction and Diversity Estimation (SPADE; Chao and Shen, 2010). In addition, bacterial OTU richness can also be estimated by fitting species abundance distribution (SAD) to parametric models. Five functions including Poisson, single exponential and finite mixtures of two, three and four exponentials were used to fit the frequency distribution of bacterial OTUs using CatchAll (version 4.0; Bunge et al., 2012), and the best model was selected following the criteria of CatchAll to estimate the OTU richness and the 95% CI.

To select the statistically best estimator, we estimated the OTU richness of a randomly selected dataset of 495 samples (covering 90% of all samples) using these non-parametric and parametric estimators, and then compared the estimates with the observed OTU richness based on the total dataset of 550 samples. The log_10_ scale of this deviation was termed as the log error of extrapolation (Dengler, 2009; Basset et al., 2012). This calculation was repeated 100 times and the estimator with the lowest mean log error of extrapolation was chose as the statistically best estimator (Dengler, 2009; Basset et al., 2012). Tukey’s Honestly Significant Difference (HSD) test was used to determine the differences of log error of extrapolation among different estimators at *P* < 0.05.

### Resampling Analyses

In order to explore the effects of environmental and spatial variation on the estimation of soil bacterial OTU richness, we conducted three resampling analyses.

#### Resampling Analysis 1

We hypothesized that the increase in sample size could reflect and capture more environmental and spatial variation and enhance the detecting ability of rare species, which consequently increasing the observed and estimated species richness even if the overall sequencing depth remained consistent. To test this, we ranked our samples according to their sequencing depth, and randomly selected 10 of the top 30 most sequenced samples and 20 of the top 30 least sequenced samples to make two comparable sub-datasets with different sample size but similar sequencing depth, and then repeated this 100 times. We performed the statistical comparisons of the number of rare OTUs (here defined as the OTUs with relative abundance lower than 0.1% across all samples), the observed and estimated OTU richness between these two sub-datasets by *t*-test (R Core Development Team, 2015).

#### Resampling Analysis 2

If the hypothesis in *Resampling analysis 1* was correct, we further examined that whether the effects of environmental and spatial variation on number of rare OTUs and the observed and estimated OTU richness for bacterial communities were different. For this purpose, we randomly selected 20 samples from the total dataset and repeated this 1,000 times. For each sub-dataset, we calculated the number of rare OTUs and the observed and estimated OTU richness, respectively. Since soil pH is previously reported as the major environmental property structuring the diversity and composition of soil bacterial communities in this plot (see Figure 1 in Barberán et al., 2015), the mean values of pairwise Euclidean distances of pH and spatial coordinates were used to assess environmental and spatial variation among samples for each sub-dataset, respectively. Multiple linear regression analyses were performed to assess the effects of environmental and spatial variation as well as the sequencing depth on the numbers of rare, observed and estimated OTUs, respectively. Then partial residuals plots were used to examine their independent effects when all other variables in the multiple regression models are statistically controlled for.

#### Resampling Analysis 3

If hypotheses in *Resampling analysis 1* and *2* were supported, we finally attempted to explore that whether these effects of environmental and spatial variation on the estimated OTU richness could be alleviated by intensive sampling scheme. To this end, we randomly selected samples from 550 soil samples according to a series of sampling levels (i.e., 10, 20, 50, 100, 150, etc.). For each sampling level, we calculated the estimated OTU richness and mean values of pairwise environmental and spatial distances as mentioned in *Resampling analysis 2*, and repeated this 1,000 times. We performed the partial Mantel test to show the correlation between the differences of estimated bacterial OTU richness and the differences of environmental or spatial variation among this 1,000 sub-datasets when other variables were controlled for. Then we plotted the changes of correlation coefficient (*r*) and statistical significance (*P*-value) along the gradient of sampling levels.

### Estimate of Soil Bacterial OTU Richness within the 50 ha Plot

We finally aimed to provide a more reliable estimate of soil bacterial OTU richness within this 50 ha FDP, because the effect of environmental variation was demonstrated to be alleviated using this intensive sampling design (see Results). The Jackknife1_ab_ estimator was found as a statistically best estimator in this study (see Results) and used for estimating the total number of soil bacterial OTU richness. Although singletons were necessary for the calculation of Jackknife1_ab_ estimator (Burnham and Overton, 1979), they were removed during sequences processing in previous study (Barberán et al., 2015). Therefore, the number of singletons was re-estimated based on the number of doubletons, tripletons and quadruplets following the recently developed method (Chiu and Chao, 2016).

### Environmental Heterogeneity Equation (EHE)

In this study, we hypothesized that increasing sampling effort can capture more environmental variation and thus enhance the probability of detecting a wider gradient of environmental properties (i.e., a greater difference between maximum and minimum values), which representing a larger niche range that available for more species to coexist. Indeed, our results demonstrated that the soil bacterial species richness was significantly affected by and positively related to the environmental variation of soil pH, while intensive sampling scheme could alleviate this effect (see Results). This highlights the importance of measuring how the captured environmental gradient responses to the sample size and determining the appropriate sample size.

For this purpose, we randomly selected sub-datasets from all 550 soil samples with a step-length of 10 samples (i.e., 10, 20, 30, etc.), and repeated this 100 times. We calculated the averages of environmental gradient (i.e., difference between maximum and minimum values of soil pH) for every sampling levels and plotted them against the sample size and fitted a curve (“nls” function in R) named EHE according to the power law function, *E* = *cS^z^*, to describe the relationship between the sample size (*S*) and the captured environmental gradient (*E*). Furthermore, we randomly reduced the total dataset to 200, 100, and 50 samples, respectively, to test whether this EHE is sensitive to the total dataset. Additionally, we simulated a more homogenous (pH range ≤ 1) dataset of 550 samples with the same mean pH value (pH = 5.8; Supplementary Table S1) of original dataset to test whether this EHE is sensitive to the extent of environmental heterogeneity.

The tangent slope of this EHE represents the derivative of EHE, suggesting that the captured environmental heterogeneity is asymptotic to the upper bound value when the tangent slope is closed to 0. Therefore, we finally explored the changes of tangent slope across sample size to determine an approximate sample size for broadly capturing the environmental gradient and estimating the bacterial species richness within habitat.

## Results

### Observed OTU Richness and the Best Estimator of Soil Bacterial OTU Richness

**FIGURE 1:**
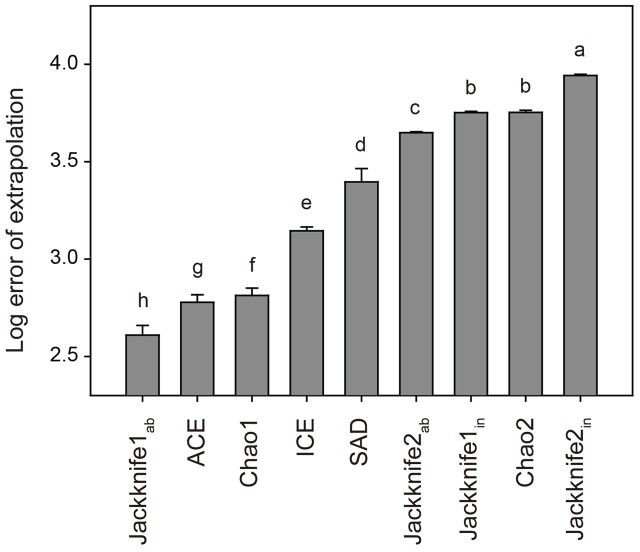
**Log error of extrapolation of different estimators for bacterial OTU richness.** Error bars are the standard deviations of the 100 randomly selected datasets and letters refer to Tukey’s HSD test grouping. See Section “Materials and Methods” for the abbreviations of estimators.

In total, 33,242 bacterial OTUs were identified at the 97% sequence similarity level across all the 550 soil samples. The number of bacterial OTUs per sample varied from 794 to 3,632, with an average of 2,578 ± 431 (mean ± SD). Results of Tukey’s HSD test showed that non-parametric Jackknife1_ab_ estimator was statistically the best for estimating the soil bacterial OTU richness, with the lowest mean log error of extrapolation among all the estimators (**Figure 1**). The advantage of Jackknife estimator is its assumption of inequality in the detection probabilities among species, while other non-parametric estimators suppose that species can be detected equally (Burnham and Overton, 1979). This advantage meets the fact that the detecting probability of rare OTUs is lower than that of abundant OTUs even though the high-throughput sequencing technologies are applied (Nemergut et al., 2011; Lynch and Neufeld, 2015). Moreover, the Jackknife estimator has been shown to perform well in previous simulation studies (Pollock and Otto, 1983; Norris and Pollock, 1996) and the Jackknife1 estimator was found to perform better than the Jackknife2 estimator with respect to the precision of species richness estimation (Palmer, 1990, 1991; Walther and Morand, 1998).

### Effects of Environmental and Spatial Variation on Estimating Soil Bacterial OTU Richness

**FIGURE 2:**
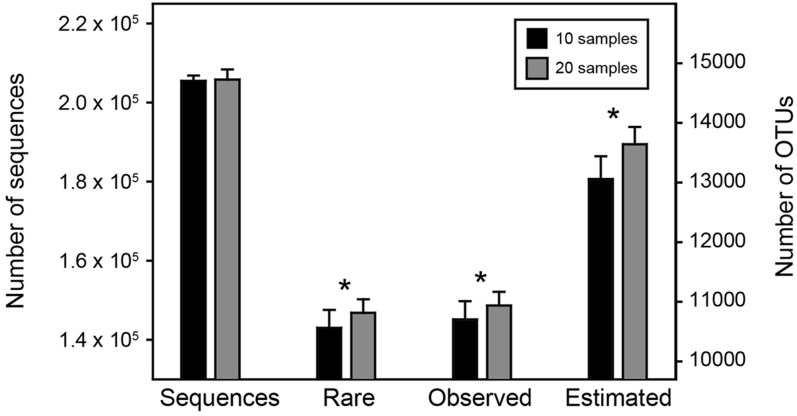
**Statistical comparisons of two sub-datasets in *Resampling analysis 1*.** The number of sequences, rare, observed and estimated OTUs between the two sub-datasets were compared by *t*-test. The asterisks above bars indicate significant difference at *P* < 0.05.

Among the 1,000 replicates in *Resampling analysis 1*, the overall sequencing depths of the two sub-datasets (i.e., less sample size with more sequences per sample vs. greater sample size with less sequences per sample) remained consistent (205,446 ± 1,371 vs. 205,815 ± 2,558, *t*-test, *P* = 0.21). However, the number of rare OTUs as well as the observed and estimated OTU richness were significantly higher (*P* < 0.05) in the sub-dataset with greater sample size (**Figure 2**). This result demonstrated our hypothesis that with similar number of microbial individuals (i.e., sequences), increasing sample size could significantly enhance the detecting ability of rare OTUs, suggesting that capturing more of the environmental and spatial variation would obtain higher observed and estimated OTU richness.

**FIGURE 3:**
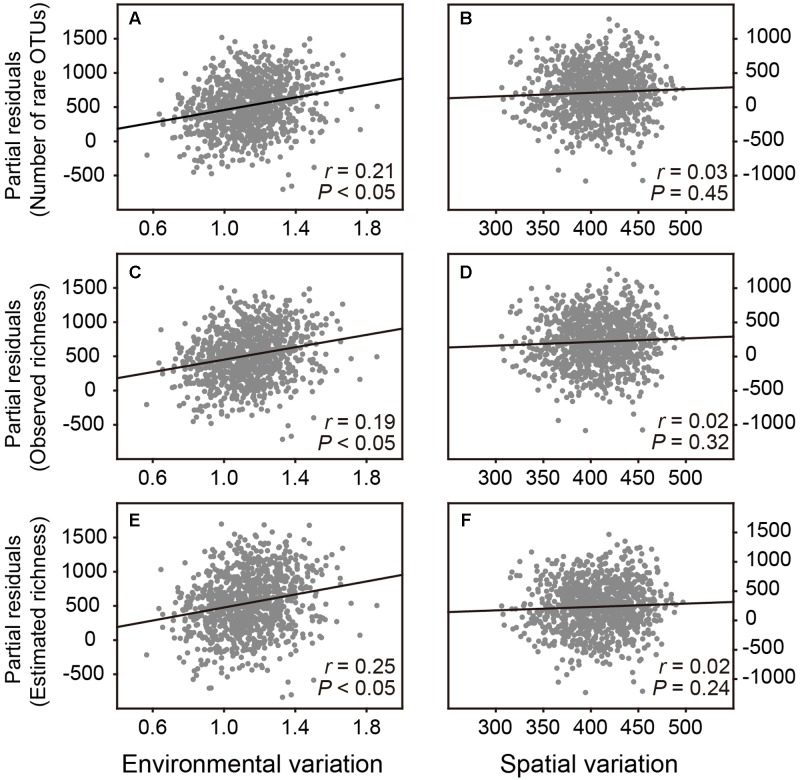
**Partial residuals plots show the independent effects of environmental and spatial variation on estimating the bacterial OTU richness.** These plots show the effect of a given independent variable when all others in the models are controlled for. **(A,B)** The effects of environmental and spatial variation on number of rare OTUs. **(C,D)** The effects of environmental and spatial variation on observed OTU richness. **(E,F)** The effects of environmental and spatial variation on estimated OTU richness.

Subsequently, we performed *Resampling analysis 2* to examine that whether environmental and spatial variation had different effects on estimating bacterial OTU richness. Partial residuals plots showed that when spatial variation and number of sequences in the multiple regression models were statistically controlled, environmental variation among samples revealed a significant and positive effect on the number of rare OTUs as well as the observed and estimated OTU richness of bacterial communities, without significant effect of spatial variation (**Figure 3** and Supplementary Table S2). These results implied that the diversity pattern of belowground soil bacterial communities was driven by environmental heterogeneity in the plot.

**FIGURE 4:**
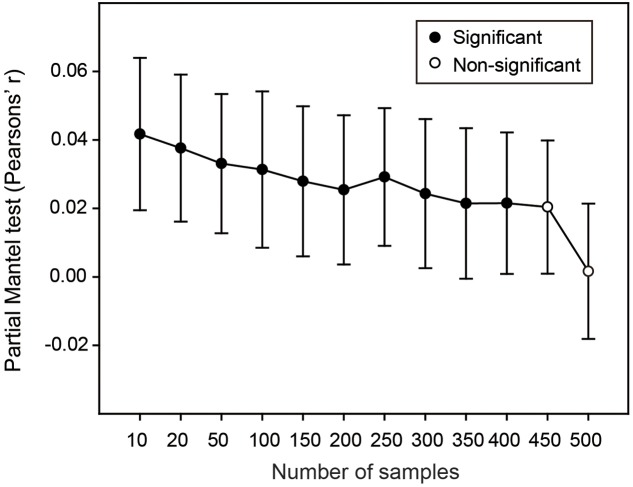
**The effects of environmental and spatial variation on estimate of bacterial OTU richness across sampling levels.** Solid and open symbols are significant and non-significant correlations, respectively. At each sampling level, the correlation between difference of estimated bacterial OTU richness and difference of environmental variation among the 1,000 sub-datasets was calculated when the differences of both spatial variation and sequencing depth were controlled for. Error bars are 95% CI.

Finally, we performed *Resampling analysis 3* to explore whether sufficient sampling could alleviate the effect of environmental variation on the estimated soil bacterial OTU richness. Partial Mantel tests along the gradient of sampling levels showed that, when the sampling levels were lower than 450, the differences of estimated bacterial OTU richness among the 1,000 replicates were significantly and positively correlated with the corresponding differences of environmental variation (**Figure 4**). Expectedly, the correlation coefficient decreased with the increase of sampling size and became non-significant when the sampling levels were higher than 450 (**Figure 4**), indicating that the effect of environmental variation on estimating bacterial OTU richness could be markedly alleviated by intensive sampling in this study.

### Estimate of Soil Bacterial OTU Richness within the Plot

Since the non-parametric Jackknife1_ab_ estimator has the merit of providing a lower-bound estimate of species richness (Gotelli and Colwell, 2001), we finally predicted that the most likely lower-bound estimate of bacterial OTU richness was 42,866 (95% CI = 42,676, 43,063) in the entire 50 ha plot.

### Building the EHE

**FIGURE 5:**
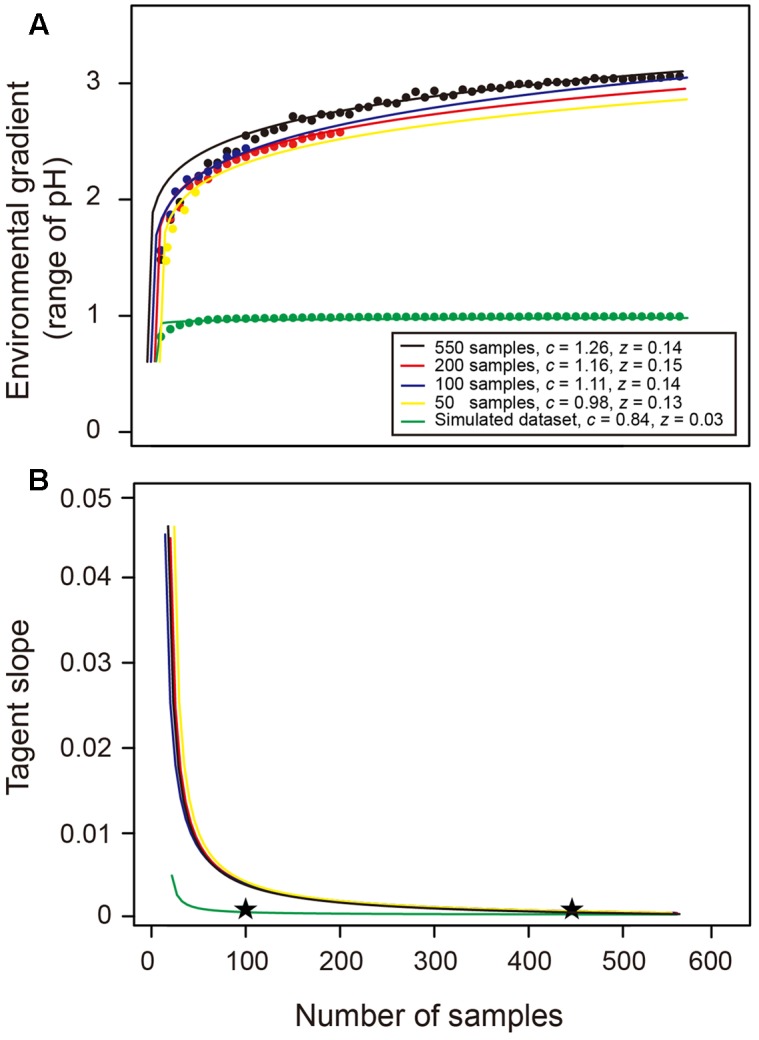
**Environmental heterogeneity equation (EHE) and the change of its tangent slope across sample sizes.** **(A)** Theses data were fitted by a power law function and these EHE describe the relationship between number of samples and captured environmental gradient (i.e., range of soil pH in this study). Black, red, blue, and yellow lines denote the EHE when the total dataset is 550, 200, 100, and 50 samples, respectively. Green line describes the EHE of our simulated homogenous dataset with narrower pH range. The *c* and *z* values denote the coefficients of power law EHE (*E* = *cS^z^*). **(B)** The tangent slopes across sample sizes represent the derivative of EHE. Stars indicated the approximate sample sizes when the slope was closed to 0.

Consistent with our hypothesis, the EHE suggests that more environmental variation could be captured with the increase in sampling effort, and thus a wider environmental gradient (i.e., greater pH range) could be detected (**Figure 5A**). A power law function was fitted to the data points of 550 samples as a specific EHE_550_ (i.e., black line in **Figure 5A**), revealing that the relationship between sample size (*S*) and captured environmental gradient (*E*) followed *E* = 1.26 × *S*^0.14^. The tangent slope of this EHE_550_ was closed to 0 when the sample size was greater than about 450 (**Figure 5B**), suggesting that the captured environmental gradient was asymptotic to the upper bound value (**Figure 5A**). Similar coefficients of the EHE and trend of their tangent slopes were found among different total datasets with 200, 100, and 50 samples, implying that the EHE of a given region is insensitive to the number of samples in total collected dataset (**Figure 5**). Comparatively, the *z* value of the EHE using a simulated more homogenous (pH gradient ≤ 1) dataset was much smaller, and its tangent slope decreased dramatically, showing that only an approximate sample size of 100 was sufficient to capture the environmental gradient in this homogenous habitat (**Figure 5**). These results suggested that the EHE could well describe the environmental heterogeneity of a given habitat, and serve as a decision-tool to determine the appropriate sample size for broadly capturing the environmental gradient and thus providing a reliable estimate of bacterial species richness.

## Discussion

Estimating the microbial species richness has always been a scientific question of great interest. Answering this question is challenging but critical in assessing biodiversity on Earth and its contribution to ecosystem function and stability. By using the scaling laws of biodiversity that is originally found in communities of plants and animals, a recent study has addressed this question based on the sequencing datasets that collected across different ecosystems, and has predicted that there are one trillion microbial species on Earth (Locey and Lennon, 2016). However, this estimate may be renewed since it is expected that the estimate of microbial species richness will keep on rising in the future due to the rapid improvement of sequencing depth and reads length (Bunge et al., 2014). Additionally, although the importance of environmental heterogeneity and geographical distance in structuring soil bacterial communities has been demonstrated in microbial biogeographic studies (Fierer and Jackson, 2006; Green and Bohannan, 2006), how they affect the estimates of bacterial species richness has rarely been explored. Therefore, instead of conducting ultra-deep sequencing to provide a more accurate estimate, in this study, we focus on the potential effects of environmental and spatial variation on estimating the total soil bacterial species richness within a given region of 50 ha FDPs, which is largely ignored in previous studies.

By performing resampling analyses to reflect and control the environmental and spatial variation, our findings demonstrate that the increase in sample size can significantly enhance the detection of rare species when holding the total sequencing depth consistent, and therefore increase the observed and estimated species richness. It should be noted that there is a tradeoff between the sample size and the number of sequences per sample for a given total sequencing depth. Specifically, a greater sample size is capable of capturing more of the environmental and spatial variation and detecting more rare species within a given habitat, while the increase in sample size will lead to a reduction in average sequencing depth per sample and a decrease in rare and observed species richness that are detected in each sample. We assume that there is an optimal balance between the sample size and sequencing depth per sample for this tradeoff to obtain the greatest number of rare species and the observed and estimated species richness, although further studies are still needed. Additionally, despite that our work emphasize the importance of comprehensively considering the effects of environmental and spatial variation in soils, previous studies have showed that deep-sequencing a single sample can uncover most of the microbial OTUs in oceans across temporal (Caporaso et al., 2012) and spatial scales (Gibbons et al., 2013). This is likely attributed to the hydrological connections between oceans and the lower turnover rate of microbial taxa in ocean rather than in soil (Zinger et al., 2014), suggesting that the strength of these effects of environmental and spatial variation is dependent on the ecosystem characteristics.

Our resampling analyses further reveal that when the sample size and sequencing depth were statistically controlled, the estimate of bacterial OTU richness is significantly higher with increasing environmental variation among samples, while there is no significant correlation between the estimate and spatial variation. This finding is in line with the previously reported results that soil bacterial diversity and community composition is strongly associated with soil pH variation but weakly correlated with spatial distance in this 50 ha forest plot (Barberán et al., 2015), supporting that environmental filtering is the main mechanism driving the community assembly of soil bacterial communities (Horner-Devine et al., 2004; Ranjard et al., 2013). It has been reported that bacteria can disperse easily within a habitat (Horner-Devine et al., 2004) but is often sensitive to different conditions of soil properties (Fierer et al., 2009; Angel et al., 2010; Rousk et al., 2010), leading to an environment-dependent pattern of diversity and composition of bacterial communities. Thus, higher environmental variation among samples can broaden the niche space that soil bacteria can occupy, especially for rare bacterial species with narrow niche breadth (Barberán et al., 2014; Lynch and Neufeld, 2015), and further provide more resource availability for more diverse bacterial taxa to coexist. In summary, our results suggest that the sampling strategy for estimating soil bacterial species richness should be able to broadly capture the full range of environmental gradient within the habitat.

We finally suppose that the effect of environmental variation can be alleviated by intensive sampling, and our resampling analyses demonstrate this expectation by revealing that the correlation coefficient between differences of environmental variation and differences of estimates decrease and become non-significant with the increase in sampling size. This finding implies that the estimates of soil bacterial species richness in previous studies with limited sample size are largely influenced by the effect of environmental variation and thus underestimated. We suppose that the re-estimated bacterial species richness of the same sites/regions with greater sample size will possibly be higher because more of the environmental variation can be captured. Thus, mathematically quantifying the minimum sample size to alleviate the effect of environmental variation should receive careful attention because it serves as a baseline for designing the sampling and sequencing scheme before estimating soil bacterial species richness. We believe that this value could be a function of area, latitude, environmental and topographical heterogeneity, spatial configuration and community evenness, etc., and fitting this model based on the metadata worldwide will provide a promising opportunity to fill this gap. In this study, we have built an EHE according to the power law function to measure the extent of environmental heterogeneity of a given region by clearly showing the relationship between the sample size and the captured environmental gradient. Specifically, this EHE is insensitive to the total dataset and the environmental heterogeneity can be assessed by single (e.g., pH in this study) or multiple (e.g., principal component) environmental variables. Therefore, *a prior* measurement of environmental properties using dozens of samples is sufficient to build this EHE, and we recommend that this EHE is a decision-tool to determine the appropriate sample size for the studies of microbial biogeography and species richness estimation.

The remarkable dataset we used in this study represents a good beginning to successfully alleviate the effect of environmental variation on estimating bacterial species richness, and thus ensures a more reliable and comprehensive assessment of biodiversity and distribution pattern of belowground soil bacterial communities in the entire 50 ha plot (Barberán et al., 2015). Similar initiatives are worthy to be implemented in other FDPs around the word to better identify generalizable patterns and interactions between aboveground and belowground communities in the most complex terrestrial habitat, using the sampling and sequencing scheme in current study (Barberán et al., 2015) as a foundation.

## Conclusion

We have highlighted the significant effect of environmental variation on the estimation of soil bacterial species richness. Further, we have demonstrated that this effect can be markedly alleviated by the intensive sampling scheme, and provided a more reliable and accurate estimate of bacterial OTU richness within a 50 ha FDP. Finally, we have built an EHE as a decision-tool for the sampling design. Except for offering a better description of soil microbial biodiversity, our work has made an important attempt to link the inherent environmental variation to the estimation of soil bacterial species richness within a given area, explaining one of the underlying mechanisms of why the previous assessment of local diversity highly varies in a habitat. Since the measurement of diversity is a starting point for further inquiry of ecological mechanisms (Shade, 2017), this study advances our understanding toward a better sampling strategy for a more reliable and accurate estimation by considering the importance of environmental heterogeneity in maintaining or changing microbial diversity in natural ecosystems.

## Author Contributions

WS framed research questions. YC, PJ, JK, LH, JL, MC, PW, and BL analyzed the data. YC, JK, PJ, and WS wrote the first draft of the manuscript and all authors contributed to discussing the results and editing the manuscript.

## Conflict of Interest Statement

The authors declare that the research was conducted in the absence of any commercial or financial relationships that could be construed as a potential conflict of interest.

## Footnotes

1 http://www.forestgeo.si.edu/

2 http://figshare.com/articles/Soil_microbial_communities_Barro_Colorado/1449286

## References

[B1] Angel R. Soares M. I. M. Ungar E. D. Gillor O. (2010). Biogeography of soil archaea and bacteria along a steep precipitation gradient. *ISME J.* 4 553–563. 10.1038/ismej.2009.136 20033070

[B2] Barberán A. McGuire K. L. Wolf J. A. Jones F. A. Wright S. J. Turner B. L. (2015). Relating belowground microbial composition to the taxonomic, phylogenetic, and functional trait distributions of trees in a tropical forest. *Ecol. Lett.* 18 1397–1405. 10.1111/ele.12536 26472095

[B3] Barberán A. Ramirez K. S. Leff J. W. Bradford M. A. Wall D. H. Fierer N. (2014). Why are some microbes more ubiquitous than others? Predicting the habitat breadth of soil bacteria. *Ecol. Lett.* 17 794–802. 10.1111/ele.12282 24751288

[B4] Basset Y. Cizek L. Cuénoud P. Didham R. K. Guilhaumon F. Missa O. (2012). Arthropod diversity in a tropical forest. *Science* 338 1481–1484. 10.1126/science.1226727 23239740

[B5] Bates S. T. Cropsey G. W. Caporaso J. G. Knight R. Fierer N. (2011). Bacterial communities associated with the lichen symbiosis. *Appl. Environ. Microbiol.* 77 1309–1314. 10.1128/AEM.02257-10 21169444 PMC3067232

[B6] Bell T. Newman J. A. Silverman B. W. Turner S. L. Lilley A. K. (2005). The contribution of species richness and composition to bacterial services. *Nature* 436 1157–1160. 10.1038/nature03891 16121181

[B7] Bohannan B. J. Hughes J. (2003). New approaches to analyzing microbial biodiversity data. *Curr. Opin. Microbiol.* 6 282–287. 10.1016/S1369-5274(03)00055-9 12831905

[B8] Bunge J. Willis A. Walsh F. (2014). Estimating the number of species in microbial diversity studies. *Ann. Rev. Stat. Appl.* 1 427–445. 10.1146/annurev-statistics-022513-115654

[B9] Bunge J. Woodard L. Böhning D. Foster J. A. Connolly S. Allen H. K. (2012). Estimating population diversity with CatchAll. *Bioinformatics* 28 1045–1047. 10.1128/mBio.00260-11.Behnke 22333246 PMC3315724

[B10] Burnham K. P. Overton W. S. (1979). Robust estimation of population size when capture probabilities vary among animals. *Ecology* 60 927–936. 10.2307/1936861

[B11] Caporaso J. G. Paszkiewicz K. Field D. Knight R. Gilbert J. A. (2012). The Western English Channel contains a persistent microbial seed bank. *ISME J.* 6 1089–1093. 10.1038/ismej.2011.162 22071345 PMC3358019

[B12] Chao A. (1984). Non–parametric estimation of the number of classes in a population. *Scand. J. Stat.* 11 265–270. 10.2307/4615964

[B13] Chao A. (1987). Estimating the population size for capture–recapture data with unequal catchability. *Biometrics* 43 783–791. 10.2307/2531532 3427163

[B14] Chao A. Lee S. M. (1992). Estimating the number of classes via sample coverage. *J. Am. Stat. Assoc.* 87 210–217. 10.1080/01621459.1992.10475194

[B15] Chao A. Shen T. J. (2010). *Program SPADE (Species Prediction And Diversity Estimation). Program and User’s Guide.* Available at: http://chao.stat.nthu.edu.tw

[B16] Chiu C. H. Chao A. (2016). Estimating and comparing microbial diversity in the presence of sequencing errors. *PeerJ* 4:e1634 10.7717/peerj.1634 PMC4741086 26855872

[B17] Condit R. Ashton P. S. Baker P. Bunyavejchewin S. Gunatilleke S. Gunatilleke N. (2000). Spatial patterns in the distribution of tropical tree species. *Science* 288:1414 10.1126/science.288.5470.1414 10827950

[B18] Connell J. H. (1978). Diversity in tropical rain forests and coral reefs. *Science* 199 1302–1303. 10.1126/science.199.4335.1302 17840770

[B19] Curtis T. P. Sloan W. T. Scannell J. W. (2002). Estimating prokaryotic diversity and its limits. *Proc. Natl. Acad. Sci. U.S.A.* 99 10494–10499. 10.1073/pnas.142680199 12097644 PMC124953

[B20] Dengler J. (2009). Which function describes the species–area relationship best? A review and empirical evaluation. *J. Biogeogr.* 36 728–744. 10.1111/j.1365-2699.2008.02038.x

[B21] Fierer N. Jackson R. B. (2006). The diversity and biogeography of soil bacterial communities. *Proc. Natl. Acad. Sci. U.S.A.* 103 626–631. 10.1073/pnas.0507535103 16407148 PMC1334650

[B22] Fierer N. Strickland M. S. Liptzin D. Bradford M. A. Cleveland C. C. (2009). Global patterns in belowground communities. *Ecol. Lett.* 12 1238–1249. 10.1111/j.1461-0248.2009.01360.x 19674041

[B23] Gevers D. Cohan F. M. Lawrence J. G. Spratt B. G. Coenye T. Feil E. J. (2005). Re-evaluating prokaryotic species. *Nat. Rev. Microbiol.* 3 733–739. 10.1038/nrmicro1236 16138101

[B24] Gibbons S. M. Caporaso J. G. Pirrung M. Field D. Knight R. Gilbert J. A. (2013). Evidence for a persistent microbial seed bank throughout the global ocean. *Proc. Natl. Acad. Sci. U.S.A.* 110 4651–4655. 10.1073/pnas.1217767110 23487761 PMC3607043

[B25] Gotelli N. J. Colwell R. K. (2001). Quantifying biodiversity: procedures and pitfalls in the measurement and comparison of species richness. *Ecol. Lett.* 4 379–391. 10.1046/j.1461-0248.2001.00230.x

[B26] Green J. Bohannan B. J. M. (2006). Spatial scaling of microbial biodiversity. *Trends Ecol. Evol.* 21 501–507. 10.1016/j.tree.2006.06.012 16815589

[B27] Groom M. J. Meffe G. K. Carroll C. R. (2006). *Principles of Conservation Biology.* Sunderland, MA: Sinauer Associates.

[B28] Hogberg P. Nordgren A. Buchmann N. Taylor A. F. S. Ekblad A. Hogberg M. N. (2001). Large-scale forest girdling shows that current photosynthesis drives soil respiration. *Nature* 411 789–792. 10.1038/35081058 11459055

[B29] Hong S. H. Bunge J. Jeon S. O. Epstein S. S. (2006). Predicting microbial species richness. *Proc. Natl. Acad. Sci. U.S.A.* 103 117–122. 10.1073/pnas.0507245102 16368757 PMC1324986

[B30] Horner-Devine M. C. Lage M. Hughes J. B. Bohannan B. J. (2004). A taxa–area relationship for bacteria. *Nature* 432 750–753. 10.1038/nature03073 15592412

[B31] Hubbell S. P. (2001). *A Unified Neutral Theory of Biodiversity and Biogeography.* Princeton, NJ: Princeton University Press.

[B32] Hughes J. B. Hellmann J. J. Ricketts T. H. Bohannan B. J. (2001). Counting the uncountable: statistical approaches to estimating microbial diversity. *Appl. Environ. Microbiol.* 67 4399–4406. 10.1128/AEM.67.10.4399 11571135 PMC93182

[B33] John R. Dalling J. W. Harms K. E. Yavitt J. B. Stallard R. F. (2007). Soil nutrients influence spatial distributions of tropical tree species. *Proc. Natl. Acad. Sci. U.S.A.* 104 864–869. 10.1073/pnas.0604666104 17215353 PMC1783405

[B34] Kemp P. F. Aller J. Y. (2004). Bacterial diversity in aquatic and other environments: what 16S rDNA libraries can tell us. *FEMS Microbiol. Ecol.* 47 161–177. 10.1016/S0168-6496(03)00257-5 19712332

[B35] Kowalchuk G. A. Stephen J. R. (2001). Ammonia–oxidizing bacteria: a model for molecular microbial ecology. *Annu. Rev. Microbiol.* 55 485–529. 10.1146/annurev.micro.55.1.485 11544365

[B36] Lee S. M. Chao A. (1994). Estimating population size via sample coverage for closed capture–recapture models. *Biometrics* 50 88–97. 10.2307/2533199 19480084

[B37] Locey K. J. Lennon J. T. (2016). Scaling laws predict global microbial diversity. *Proc. Natl. Acad. Sci. U.S.A.* 113 5970–5975. 10.1073/pnas.1521291113 27140646 PMC4889364

[B38] Lynch M. D. Neufeld J. D. (2015). Ecology and exploration of the rare biosphere. *Nat. Rev. Microbiol.* 13 217–229. 10.1038/nrmicro3400 25730701

[B39] MacArthur R. H. Wilson E. O. (1967). *The Theory of Island Biogeography.* Princeton, NJ: Princeton University Press.

[B40] Maestre F. T. Quero J. L. Gotelli N. J. Escudero A. Ochoa V. Delgado-Baquerizo M. (2012). Plant species richness and ecosystem multifunctionality in global drylands. *Science* 335 214–218. 10.1126/science.1215442 22246775 PMC3558739

[B41] Martiny J. B. H. Eisen J. A. Penn K. Allison S. D. Horner-Devine M. C. (2011). Drivers of bacterial (-diversity depend on spatial scale. *Proc. Natl. Acad. Sci. U.S.A.* 108 7850–7854. 10.1073/pnas.1016308108 21518859 PMC3093525

[B42] May R. M. (1988). How many species on earth? *Science* 241 1441–1449. 10.1126/science.241.4872.1441 17790039

[B43] Nemergut D. R. Dinana R. Costello E. K. Hamady M. Lozupone C. Jiang L. (2011). Global patterns in the biogeography of bacterial taxa. *Environ. Microbiol.* 13 135–144. 10.1111/j.1462-2920.2010.02315.x 21199253 PMC5834236

[B44] Norris J. L. Pollock K. H. (1996). Non-parametric MLE under two closed capture-recapture models with heterogeneity. *Biometrics* 52 639–649. 10.2307/2532902

[B45] Palmer M. W. (1990). The estimation of species richness by extrapolation. *Ecology* 71 1195–1198. 10.2307/1937387

[B46] Palmer M. W. (1991). Estimating species richness: the second-order jackknife reconsidered. *Ecology* 72 1512–1513. 10.2307/1941127

[B47] Pollock K. H. Otto M. C. (1983). Robust estimation of population size in closed animal population from capture-recapture experiments. *Biometrics* 39 1035–1049. 10.2307/2531337 6671118

[B48] R Core Development Team (2015). *R: A Language and Environment for Statistical Computing.* Vienna: R Foundation for Statistical Computing.

[B49] Ramette A. Tiedje J. M. (2007). Multiscale responses of microbial life to spatial distance and environmental heterogeneity in a patchy ecosystem. *Proc. Natl. Acad. Sci. U.S.A.* 104 2761–2766. 10.1073/pnas.0610671104 17296935 PMC1815255

[B50] Ranjard L. Dequitedt S. Prevost-Boure N. C. Thioulouse J. Saby N. P. A. Lelievre M. (2013). Turnover of soil bacterial diversity driven by wide–scale environmental heterogeneity. *Nat. Commun.* 4:1434 10.1038/ncomms2431 23385579

[B51] Rillig M. C. Mummey D. L. (2006). Mycorrhizas and soil structure. *New Phytol.* 171 41–53. 10.1111/j.1469-8137.2006.01750.x 16771981

[B52] Roesch L. F. W. Fulthorpe R. R. Riva A. Casella G. Hadwin A. K. M. Kent A. D. (2007). Pyrosequencing enumerates and contrasts soil microbial diversity. *ISME J.* 1 283–290. 10.1038/ismej.2007.53 18043639 PMC2970868

[B53] Rosselló-Mora R. Amann R. (2001). The species concept for prokaryotes. *FEMS Microbiol. Rev.* 25 39–67. 10.1111/j.1574-6976.2001.tb00571.x 11152940

[B54] Rousk J. Baath E. Brookes P. C. Lauber C. L. Lozupone C. Caporaso J. G. (2010). Soil bacterial and fungal communities across a pH gradient in an arable soil. *ISME J.* 4 1340–1351. 10.1038/ismej.2010.58 20445636

[B55] Schloss P. D. Handelsman J. (2006). Toward a census of bacteria in soil. *PLoS Comput. Biol.* 2:e92 10.1371/journal.pcbi.0020092 PMC1513271 16848637

[B56] Shade A. (2017). Diversity is the question, not the answer. *ISME J.* 11 1–6. 10.1038/ismej.2016.118 27636395 PMC5421358

[B57] Slik J. W. F. Arroyo-Rodríguez V. Aiba S. I. Alvarez-Loayza P. Alves L. F. Ashton P. (2015). An estimate of the number of tropical tree species. *Proc. Natl. Acad. Sci. U.S.A.* 112 7472–7477. 10.1073/pnas.1423147112 26034279 PMC4475970

[B58] Stevens G. C. (1989). The latitudinal gradient in geographical range: how so many species coexist in the tropics. *Am. Nat.* 133 240–256. 10.1086/284913

[B59] Stork N. E. McBroom J. Gely C. Hamilton A. J. (2015). New approaches narrow global species estimates for beetles, insects, and terrestrial arthropods. *Proc. Natl. Acad. Sci. U.S.A.* 112 7519–7523. 10.1073/pnas.1502408112 26034274 PMC4475949

[B60] Walther B. A. Morand S. (1998). Comparative performance of species richness estimation methods. *Parasitology* 116 395–405. 10.1017/S0031182097002230 9585941

[B61] Whitman W. B. Coleman D. C. Wiebe W. J. (1998). Prokaryotes: the unseen majority. *Proc. Natl. Acad. Sci. U.S.A.* 95 6578–6583. 10.1073/pnas.95.12.6578 9618454 PMC33863

[B62] Yarza P. Yilmaz P. Pruesse E. Glöckner F. O. Ludwig W. Schleifer K. H. (2014). Uniting the classification of cultured and uncultured bacteria and archaea using 16S rRNA gene sequences. *Nat. Rev. Microbiol.* 12 635–645. 10.1038/nrmicro3330 25118885

[B63] Youssef N. Sheik C. S. Krumholz L. R. Najar F. Z. Roe B. A. Elshahed M. S. (2009). Comparison of species richness estimates obtained using nearly complete fragments and simulated pyrosequencing–generated fragments in 16S rRNA gene–based environmental surveys. *Appl. Environ. Microbiol.* 75 5227–5236. 10.1128/AEM.00592-09 19561178 PMC2725448

[B64] Zinger L. Boetius A. Ramette A. (2014). Bacterial taxa–area and distance–decay relationships in marine environments. *Mol. Ecol.* 23 954–964. 10.1111/mec.12640 24460915 PMC4230465

