# Supplementary material for: Effect of Environmental Variation on Estimating the Bacterial Species Richness
Source: Front Microbiol. 2017 Apr 19;8:690. doi: 10.3389/fmicb.2017.00690 (PMC5395623; doi:10.3389/fmicb.2017.00690)
Supplement: Supplementary file 1 [file Table_1.DOCX]

**Supplementary Table S1 | Soil environmental variables in the BCI plot.**

| Soil environmental variables | Mean ± SD | Range | Unit |
| --- | --- | --- | --- |
| pH | 5.8 ± 0.5 | 4.6 - 7.6 |  |
| Fe | 1.7 ± 7.9 | 0 - 158.6 | mg/kg |
| K | 110 ± 71.1 | 0.5 - 487.2 | mg/kg |
| Mg | 304.1 ± 179.3 | 35.4 - 2369 | mg/kg |
| Mn | 76.5 ± 62.2 | 0.5 - 358.3 | mg/kg |
| Na | 11.3 ± 45.9 | 0 - 1009.9 | mg/kg |
| C/N | 10.5 ± 9.4 | 7.6 - 17.1 |  |
