# Supplementary material for: Effect of Environmental Variation on Estimating the Bacterial Species Richness
Source: Front Microbiol. 2017 Apr 19;8:690. doi: 10.3389/fmicb.2017.00690 (PMC5395623; doi:10.3389/fmicb.2017.00690)
Supplement: Supplementary file 2 [file Table_2.DOCX]

**Supplementary Table S2 | Results of multiple regression on the number of rare, observed and estimated OTUs.**

|  | Environmental variation | Spatial variation | Sequencing depth | *R*^2^ |
| --- | --- | --- | --- | --- |
| Rare | 0.21^***^ | 0.03 | 0.42^***^ | 0.25 |
| Observed | 0.19^***^ | 0.02 | 0.42^***^ | 0.25 |
| Estimated | 0.25^***^ | 0.02 | 0.35^***^ | 0.19 |

The standardized partial regression coefficients and *R*^2^ of the models were reported and the asterisks indicate signiﬁcant levels. ^*^*P* < 0.05, ^**^*P* < 0.01, and ^***^*P* < 0.001.
